# An innovative anti-cancer Chinese herbal formula exhibited multi-targeted efficacies in metastatic breast cancer mouse model

**DOI:** 10.1186/s13020-018-0222-9

**Published:** 2018-12-22

**Authors:** Grace Gar-Lee Yue, Julia Kin-Ming Lee, Ben Chung-Lap Chan, Hin-Fai Kwok, Sandy Wan-Heng Hoi, Daniel Man-Yuen Sze, Kwok-Pui Fung, Ping-Chung Leung, Clara Bik-San Lau

**Affiliations:** 1 Institute of Chinese Medicine, E205, Science Centre East Block, The Chinese University of Hong Kong, Shatin, New Territories, Hong Kong; 2 State Key Laboratory of Research On Bioactivities and Clinical Applications of Medicinal Plants, The Chinese University of Hong Kong, Shatin, New Territories, Hong Kong; 3 School of Biomedical Sciences, The Chinese University of Hong Kong, Shatin, New Territories, Hong Kong; 4 Department of Health Technology and Informatics, The Hong Kong Polytechnic University, Hung Hom, Kowloon, Hong Kong

**Keywords:** Metastatic breast cancer, Chinese herbal medicines, Metastasis, *Andrographis paniculata*, *Acanthopanax senticosus*, *Camellia sinensis*, *Hedyotis diffusa*

## Abstract

**Background:**

The incidence and mortality of cancer metastasis is high worldwide. Despite of the chemotherapeutic agents, many cancer patients still take traditional Chinese herbal prescriptions as adjuvant treatments. However, most of these herbal formulae/products lack of evidence-based efficacy. Based on our previous investigations on anti-tumor, anti-angiogenic, anti-metastatic, bone protective and immunomodulating activities of various Chinese herbal medicines, four constituent herbs, namely *Andrographis paniculata*, *Acanthopanax senticosus*, *Camellia sinensis*, and *Hedyotis diffusa* were eventually selected to form an innovative herbal formula.

**Methods:**

The anti-tumor efficacies of the formula were evaluated in metastatic breast cancer mice model. The bone protective and immunomodulatory effects were also assessed after formula treatment.

**Results:**

Our results showed that the breast tumor weight as well as lung and liver metastasis in mice could be reduced after herbal formula treatment for 4 weeks. The breast tumor-induced osteolysis in mice was restored by herbal formula treatment, in which the bone volume in treated mice tibia was comparable to that in the non-tumor bearing normal mice. The IL-12 level was augmented and the survival of mice with metastatic breast tumors was prolonged after treatment. Furthermore, combination of herbal formula with chemotherapeutic agent doxorubicin resulted in better anti-tumor efficacy and increased life span in tumor-bearing mice, when compared with doxorubicin alone treatment.

**Conclusions:**

In summary, our innovative Chinese herbal formula was demonstrated to possess anti-tumor, anti-metastatic and bone-protective activities in metastatic breast tumor-bearing mice. The preclinical data generated in this study would lead to the development of evidence-based supplement as adjuvant therapy for metastatic breast cancer.

**Electronic supplementary material:**

The online version of this article (10.1186/s13020-018-0222-9) contains supplementary material, which is available to authorized users.

## Background

Metastatic cancer is a cancer that has spread from the original site to other tissues and organs. Certain types of cancer are more likely to spread to certain areas than others [1]. Once cancer spreads, the improvement of prognosis continues to provide challenges for patients and healthcare providers. There are treatments for some types of metastatic cancer but not all these treatments are effective enough to control the spread of cancer as well as to relieve symptoms. Limitations of the conventional treatments over their benefits occur.

The increasing use of Chinese herbal medicines (CHM) by cancer patients and survivors can be seen worldwide [2–4]. Cancer patients used CHM to improve their physical and emotional well-beings and to reduce cancer therapy-induced toxicities [5–7]. We have previously proposed that cancer treatment approach needs to be modified from a single target and eliminative direction to a multiple target and holistic direction, which might be achieved by using Chinese herbal medicines as adjuvant therapy [8]. This anticipation has long been pursued by our research team in developing a novel Chinese herbal formula for multi-target therapy approaches [9]. We have also made attempts on establishment of cancer research platforms for modern research of herbal medicines and natural products. One of our focused area is breast cancer treatment.

Breast cancer affects approximately 1.67 million women worldwide in 2012 [10] and it is the 2nd leading causes of cancer death in US [11]. In China, estimated incidence was around 11 out of 100,000 in 2013 [12] and the incidence rate is still increasing [13]. In Chinese community, including Hong Kong, many breast cancer patients take traditional Chinese herbal prescriptions as adjuvant treatments. In view of the high incidence rate of metastatic breast cancer worldwide, the efficacies of CHM were initially investigated in our pre-clinical breast cancer research platform.

In the past decade, our research team has carried out a series of investigations on the anti-tumor activities of various Chinese medicinal herbs and their isolated compounds. Among these herbs, *Andrographis paniculata* (AP, 穿心蓮), *Acanthopanax senticosus* (AS, 刺五加), *Camellia sinensis* (CS, 綠茶), and *Hedyotis diffusa* (HD, 白花蛇舌草) were shown to have promising anti-cancer-related bioactivities. Aqueous extract of AP has been shown to potently inhibit tumor growth and metastasis in esophageal cancer mice in our previous studies [14–16]. *Acanthopanax senticosus* (AS) was investigated for its anti-tumor and immunomodulatory activities in breast cancer cells and immuno-competent mice models [17], unpublished data]. Besides, the anti-tumor, anti-metastatic and bone protective effects of *Camellia sinensis* (CS) have been elucidated thoroughly in a series of studies [18–20]. In addition, both the aqueous extract and the active component from *Hedyotis diffusa* (HD), 4-vinylphenol, have also been demonstrated for their potent anti-angiogenic activities in animal models [21]. The optimal dosages of each herb have been validated in the corresponding in vivo experiments mentioned above. Based on these results, an innovative herbal formula of these four constituent herbs with particular reference to the properties was formed using their corresponding optimal dosages, hence, the ratio of each herb in the formula was AP:AS:CS:HD = 1.7:1:1:8. This formula was designed for cancer patients as it was expected to augment and to relieve the discomfort from the conventional cancer treatment as well as prevent recurrence. The present study aimed at evaluating the efficacies of the herbal formula in our breast tumor-bearing mice model. Promising results showed the anti-metastatic effects of the formula. Furthermore, the adjuvant activities of the formula to chemotherapeutic agent doxorubicin in metastatic breast cancer mice were illustrated for the first time.

## Materials and methods

### Information of experimental design and resources

The information regarding the experimental design, statistics, and resources used in this study is attached in the minimum standards of reporting checklist (Additional file 1).

### Herbal materials and extraction

The dried herbs of *Andrographis paniculata* (AP), *Acanthopanax senticosus* (AS), *Camellia sinensis* (CS) and *Hedyotis diffusa* (HD) were purchased in a single lot from herbal suppliers in Hong Kong. Morphological and chemical authentication were accomplished in accordance with the Chinese Pharmacopoeia 2010 [22] or herbal medicine reference book (since no monograph was found in Chinese Pharmacopoeia for *Hedyotis diffusa*) [23]. The chemical profiles were compared qualitatively using thin layer chromatography (TLC) with the reference herb provided by National Institute for the Control of Pharmaceutical and Biological Products. In addition, the representative chemical markers (andrographolide and dehydroandrographolide for AP; syringin for AS; caffeine, epigallocatechin gallate for CS; asperuloside for HD) were determined quantitatively using UPLC. Authenticated voucher specimens were deposited in the museum of the Institute of Chinese Medicine, the Chinese University of Hong Kong.

Dried herbs were extracted individually under reflux using water for 1 h and the extraction were repeated once. The extracts were filtrated, centrifuged to remove undissolved particles and then freeze-dried into powder. The formula composed of 4 types of aqueous extracts of the above-mentioned herbs, with the ratio (AP:AS:CS:HD = 1.7:1:1:8). The optimal dosages of each herb were mixed (i.e. 1000 mg/kg for AP; 600 mg/kg for AS; 600 mg/kg for CS; 4800 mg/kg for HD, 7000 mg/kg in total). The mixed formula extract powder was dissolved in water for animal studies.

### UPLC-QTOF analysis of the herbal formula

#### Stock solutions

Stock solutions of syringin, asperuloside, caffeine, epigallocatechin gallate, andrographolide and dehydroandrographolide were prepared individually in distilled water at 1 mg/mL. They were stored at − 20 °C until use.

#### Preparation of standard solution

100 µL of each stock solution were mixed and marked up to 1 mL by distilled water to give a standard solution.

#### Sample preparation

140 mg of dry sample powder were dissolved in 5 mL distilled water as the sample solution.

#### UPLC-QTOF analysis

**Fig. 1:**
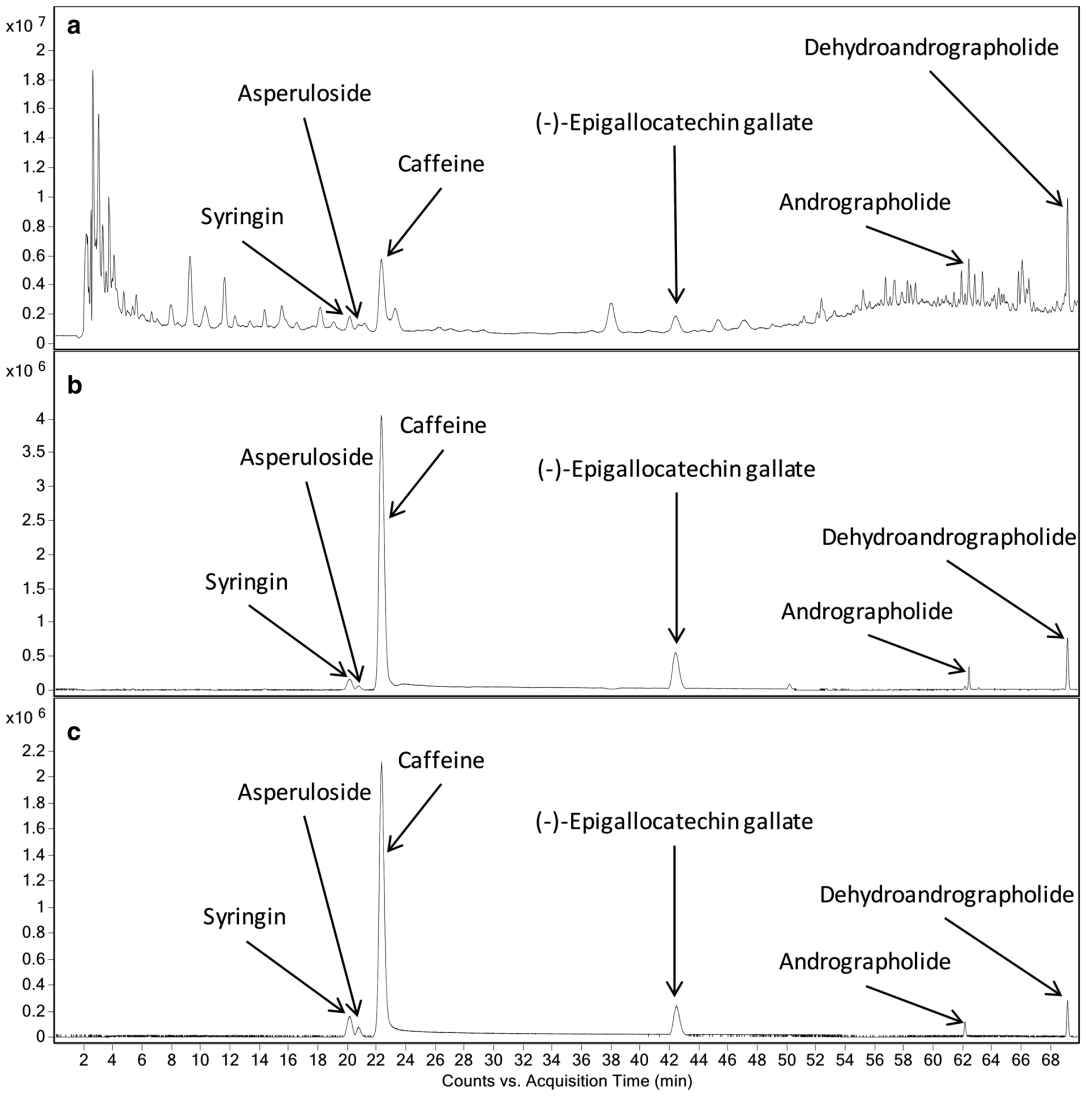
UPLC chromatograms of the herbal formula. **a** Total ion chromatogram of sample; **b** Base peak chromatogram of sample; **c** Base peak chromatogram of standard markers. 395.1318 m/z (Syringin), 437.1049 m/z (Asperuloside), 195.0874 (Caffeine), 459.0913 (Epigallocatechin gallate), 315.1956 m/z (Andrographolide) and 297.1855 m/z (Dehydroandrographolide) were extracted for the base peak chromatogram

The analysis was conducted using an Agilent 1290 UHPLC with 6530 QTOF system (CA, USA). The column used was GRACE Alltima C18, 5 µm, 4.6 × 250 mm. The chromatographic separation was conducted at room temperature under gradient conditions at a flow rate of 1 mL/min. The LC profile was as follows: Mobile phase: (A) (0.1% formic acid, in deionized and distilled water) and (B) acetonitrile; Gradient: 0–5 min, 8% B; 5–10 min, 8–10% B; 10–30 min, 10% B; 30–42 min, 10–13% B; 42–49 min, 13–18% B; 49–69 min, 18–55% B; 69–70 min, 55% B. High purity nitrogen was used as the curtain and collision gas with a flow rate of 12 L/min. The drying gas temperature was set at 350 °C, and the nebulizer pressure was set at 60 psi. Spectra were recorded in positive ion mode at spray voltage of 3500 V. The mass scan range was between 100 and 1100 m/z. Data analysis was performed using Agilent MassHunter Workstation Qualitative Analysis Software (CA, USA, version B.06.00). Syringin was determined at 395.1318 m/z [M + Na]^+^, asperuloside was determined at 437.1049 m/z [M + Na]^+^, caffeine was determined at 195.0874 m/z [M + H]^+^, epigallocatechin gallate was determined at 459.0913 m/z [M + H]^+^, andrographolide was determined at 315.1956 m/z [M-2H_2_O + H]^+^ and dehydroandrographolide was determined at 297.1855 m/z [M-2H_2_O + H]^+^. The chemical profile of the formula extract was shown as chromatograms in Fig. 1. The contents of these chemical markers in the formula extract were listed in Table 1.

**Table 1:** Contents of chemical markers (syringin, asperuloside, caffeine, epigallocatechin gallate, andrographolide and dehydroandrographolide) in formula extract

Chemical markers	Content in formula extract (%)
Syringin	0.043 ± 0.005
Asperuloside	0.044 ± 0.003
Caffeine	0.596 ± 0.018
Epigallocatechin gallate	0.817 ± 0.021
Andrographolide	0.475 ± 0.033
Dehydroandrographolide	0.115 ± 0.008

### Cell culture

Mouse breast tumor cells (4T1) were purchased from American Type Culture Collection (MD, USA) and maintained in RPMI medium. Culture media were supplemented with 10% (v/v) heat-inactivated FBS, 100 units/mL penicillin–streptomycin. The cells were incubated at 37 °C in a humidified atmosphere of 5% CO_2_. When the cells reached 80% confluence in culture flasks, trypsin–EDTA was used to remove the cells and the cells were used in experiments or reseeded in flask. Culture medium, fetal bovine serum (FBS), penicillin–streptomycin, trypsin–EDTA, were obtained from Life Technologies (NY, USA).

### Mouse breast tumor model

Female BALB/c mice (6–8 weeks old) were supplied and maintained by Laboratory Animal Services Center, the Chinese University of Hong Kong. All experimental procedures in mice complied with the standards specified by the Animal Experimentation Ethics Committee of the Chinese University of Hong Kong (Ref No. 10/051/MIS).

Mouse breast tumor cells 4T1 (4 × 10^5^) resuspended in 0.1 mL PBS, were subcutaneously inoculated at the mammary fat pad of each mouse. Treatments were initiated 8 days after tumor cell implantation and lasted for 4 weeks. After 4T1 cell implantation, the tumor-bearing mice were randomly assigned into different treatment groups (n = 12). In the pilot study, mice were orally administered with formula at 7000 mg/kg (mixed 4 herbal extract at optimal dosage); however, the efficacy of 7000 mg/kg of formula reached plateau and the solubility of the herbal formula powder was not good at such dosage (i.e. ≥ 7000 mg/kg). Hence, the testing dosage was reduced around 3-, 10-, 30-folds to 2310, 770 and 231 mg/kg, respectively. Tumor-bearing mice were orally administered with formula at 3 different dosages (L: 231 mg/kg; M: 770 mg/kg; H: 2310 mg/kg, daily) and/or treated with doxorubicin (5 mg/kg, once a week) for 4 weeks. The mixed formula powder was dissolved in distilled water. Chemotherapeutic agent doxorubicin at 5 mg/kg was dissolved in saline and administered intraperitoneally to mice. The body weights and tumor sizes of mice were measured once a week during the treatment period. At the end of treatment (8 weeks), mice were sacrificed and blood was collected. The cytokines levels in plasma isolated from mice were determined by ELISA (BD Biosciences, NJ, USA). Lungs and livers were removed for histological analysis. Tibias of mice from different groups were removed for micro-computed tomography analysis.

### Immunological responses in breast tumor-bearing mice

The immunomodulatory effects of the formula on breast tumor-bearing mice were further determined. Cells harvested from thymus, spleen and lymph nodes were subjected to population determination of T cells, regulatory T cells (Treg) or myeloid-derived suppressor cells (MDSC) by flow cytometry. In brief, the excised organs were pressed through the nylon membranes using syringe plungers and the cells were washed and blocked using PBS supplemented with 5% FBS. Cells were stained with a cocktail of fluorescence-conjugated anti-CD3, anti-CD4 and anti-CD8 IgG for T cells, or a cocktail of anti-CD4, anti-CD25 and anti-FOXP3 antibodies for regulatory T cells, or a cocktail of anti-Ly6G and anti-CD11b antibodies for MDSC. All stained cells were analyzed using a FACSCanto™ II Flow Cytometer (BD Biosciences, NJ, USA).

Lymphocytes harvested from spleens of mice were cultured ex vivo as described previously [27]. Lymphocytes were resuspended in RPMI 1640 medium, seeded in 24 well-plates at a density of 3 × 10^6^ cells/mL and incubated with phytohemagglutinin (PHA) at 10 mg/mL for 24 h. After incubation, the culture supernatants were collected and subjected to enzyme-linked immunosorbent assay (ELISA) for concentration determination of IL-2, IL-12, IFN-γ and TNF-α according to the kit protocols.

### Histological analysis

Lungs and livers were fixed in 10% buffered formalin, paraffin embedded and sectioned at 5 μm. Then the sections were stained with hematoxylin & eosin and examined and photographed as described previously [28]. Tumor burden, defined as the tumor area, was calculated from the section of the lung or liver in a double-blind manner using Image J software (NIH, USA). The section photos taking and assessment using Image J software were performed by two investigators. Tumor burden expressed as an average percentage of tumor area to lung or liver area in each treatment group.

### Micro-computed tomography (μ-CT) analysis

Tibias removed from tumor-bearing mice were scanned with a high resolution microtomographic system, μ-CT 40 (Scanco Medical AG, Switzerland). Each three-dimensional image data consisted of approximately 500 μ-CT slide image (8 μm/slide) starting from the growth plate of tibial interface and moving down the tibia. Bone volume (mm^3^) was generated from μ-CT analysis and compared with the control tibia for each animal [24].

### Statistical analysis

Data were expressed as mean + SEM. Statistical analyses and significance were analyzed by one-way ANOVA followed by post hoc test using GraphPad PRISM software version 6.0 (GraphPad Software, USA). In all comparisons, *p* < 0.05 was considered as statistically significant.

## Results

### Herbal formula treatment significantly reduced weight of breast tumors without affecting body weight

**Fig. 2:**
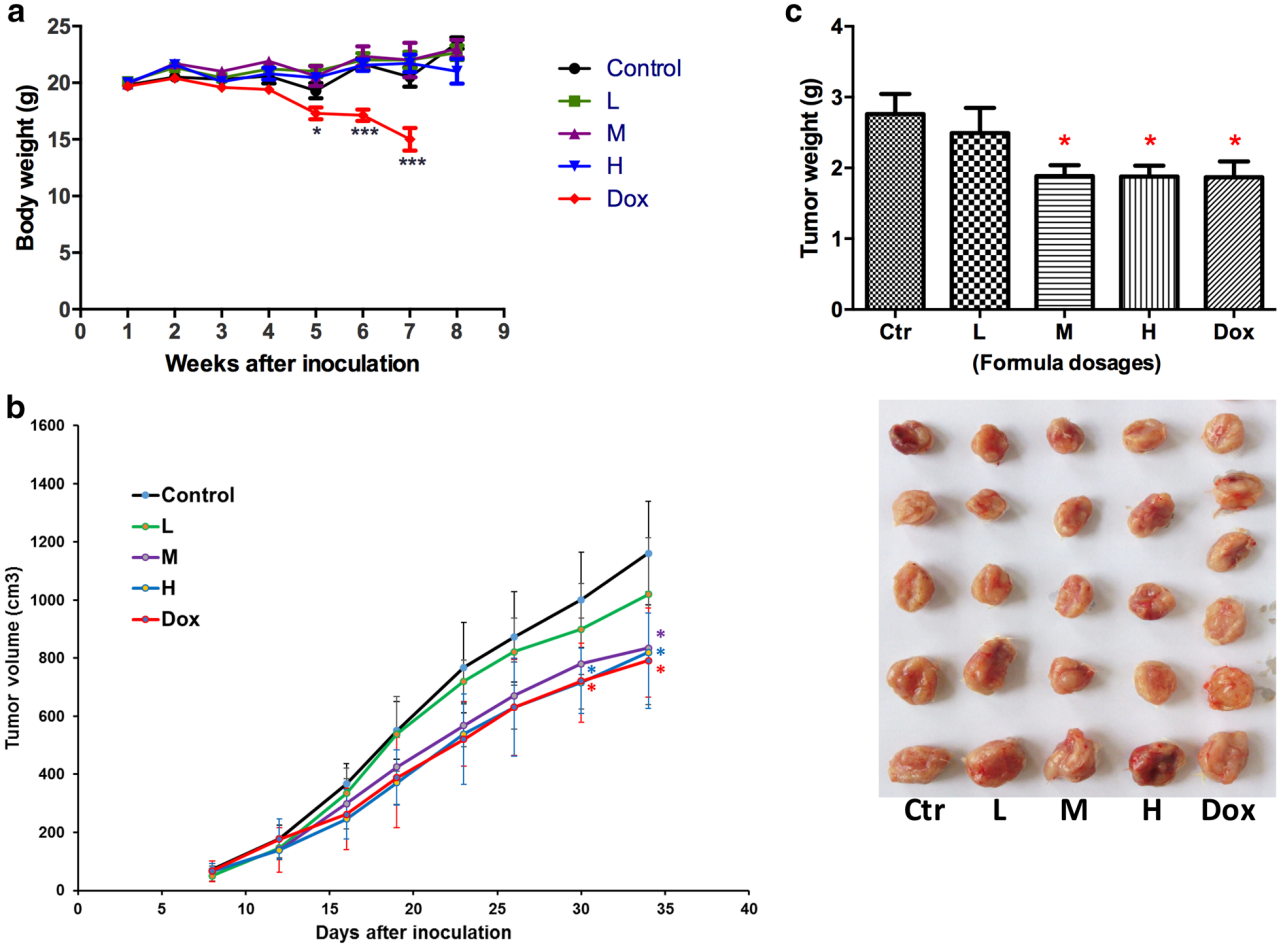
Effects of formula or doxorubicin (Dox) treatments on the 4T1 breast tumor-bearing mice. **a** Body weight in different treatment groups (L: 231 mg/kg; M: 770 mg/kg; H: 2310 mg/kg, daily). Each point represented mean ± SEM (n = 12). **b** Tumor volume of mice were recorded during treatments. **c** Effects of different treatments on final tumor weight were compared. Results were expressed as mean + SEM (n = 12). *p < 0.05, ***p < 0.005 when treatment groups compared with control group at the same time point by one-way ANOVA followed by Tukey’s multiple comparisons test. Photograph at lower panel showed the representative tumors in different treatment groups

Tumor-bearing mice were orally administered with formula at 3 different dosages (L: 231 mg/kg; M: 770 mg/kg; H: 2310 mg/kg, daily) or treated with doxorubicin (5 mg/kg, once a week) for 4 weeks. During the treatments, no body weight loss was observed in formula-treated groups while there was significant decrease in doxorubicin-treated group since the 4th week after treatment started (Fig. 2a). The tumor volume was shown to be lowered in formula-treated (M and H dosages) and doxorubicin-treated groups (Fig. 2b) when compared with untreated control group. The tumor weights of mice after treatments with formula (at M and H dosages) or doxorubicin were significantly lowered than those of untreated control mice (*p* < 0.05, Fig. 2c).

### Herbal formula treatment significantly reduced lung and liver metastasis and restored breast tumor-induced osteolysis

**Fig. 3:**
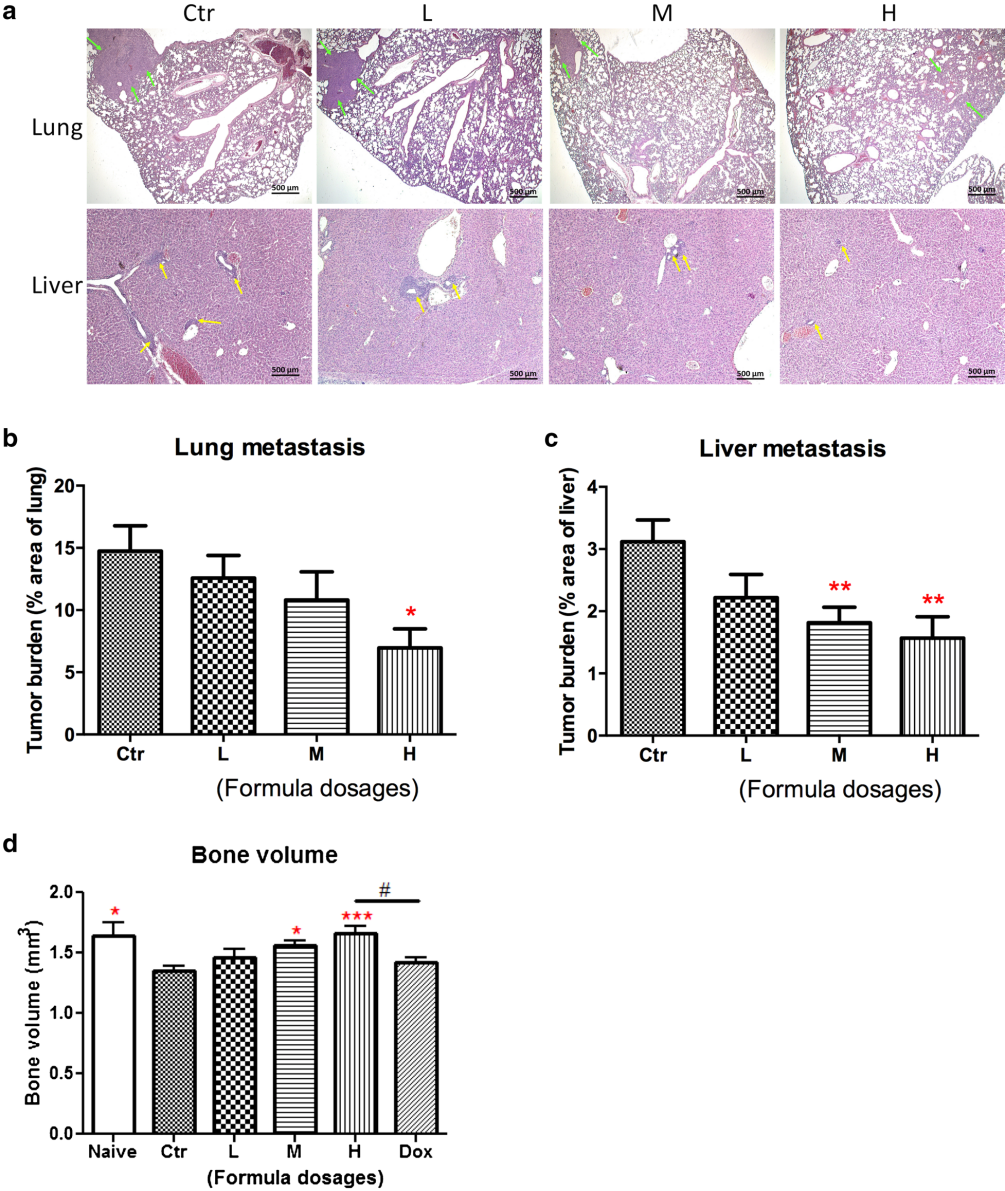
The paraffin-embedded sections of the lungs and livers were photographed and used to measure metastatic loci area and total lung or liver area. **a** Representative photographs of lungs and livers were shown. Arrows indicated the tumor nodules. The histograms showed the tumor burden in **b** lungs and **c** livers according to the tumor area as a percentage of whole lung or liver area per group (n = 12). **d** Effects of formula treatments on bone volume of tumor-bearing mice. Quantitative assessment of bone structure in mouse tibias after formula or doxorubicin treatments. The graph showed the bone volume of tibias from different groups (n = 10). Data were expressed as mean + SEM. Differences among the treated and control groups were determined by one-way ANOVA followed by Tukey’s multiple comparisons test. *p < 0.05, **p < 0.01, ***p < 0.005 as compared to control group. ^#^*p* < 0.05 when compared among treatment groups

Lungs and livers from each mouse were removed for histological analysis and assessment of lung and liver metastasis. Large tumors were found in untreated control group, while the tumor area and nodules were decreased in formula treatment groups (Fig. 3a). Tumor burden in lung was found to decrease by formula treatment (Fig. 3b). Tumor burden in lung decreased by 52.8% (from 14.74 to 6.96%) in H-dosage formula-treated group. Similar results were also found in liver metastasis. Liver metastasis was decreased significantly after formula treatments (both M- & H-dosages) (*p* < 0.01) (Fig. 3c). Tumor burden in liver decreased by 42.0% and 49.7% in M- and H-dosages formula-treated groups.

As mentioned, breast cancer cells metastasize to bone and induce severe bone destruction. For assessing the efficacy of formula treatment against breast cancer-induced bone destruction, micro-computed tomography (μ-CT) analysis was employed. Tumor-bearing mice with formula treatments showed increased bone volume in a dose-dependent manner. As shown in Fig. 3d, the bone volume (BV) in control group was 1.35 mm^3^, lost nearly 18% of BV when compared to the BV of naive group, indicating severe bone destruction in control group. In contrast, treatments with formula (at dosages M & H) resulted in remarkable increase of BV in tumor-bearing mice. No significant difference was shown between doxorubicin-treated and control groups, but there was significant difference between doxorubicin-treated and formula dosage H groups (*p* < 0.05).

### Herbal formula treatment modulated immune responses

**Fig. 4:**
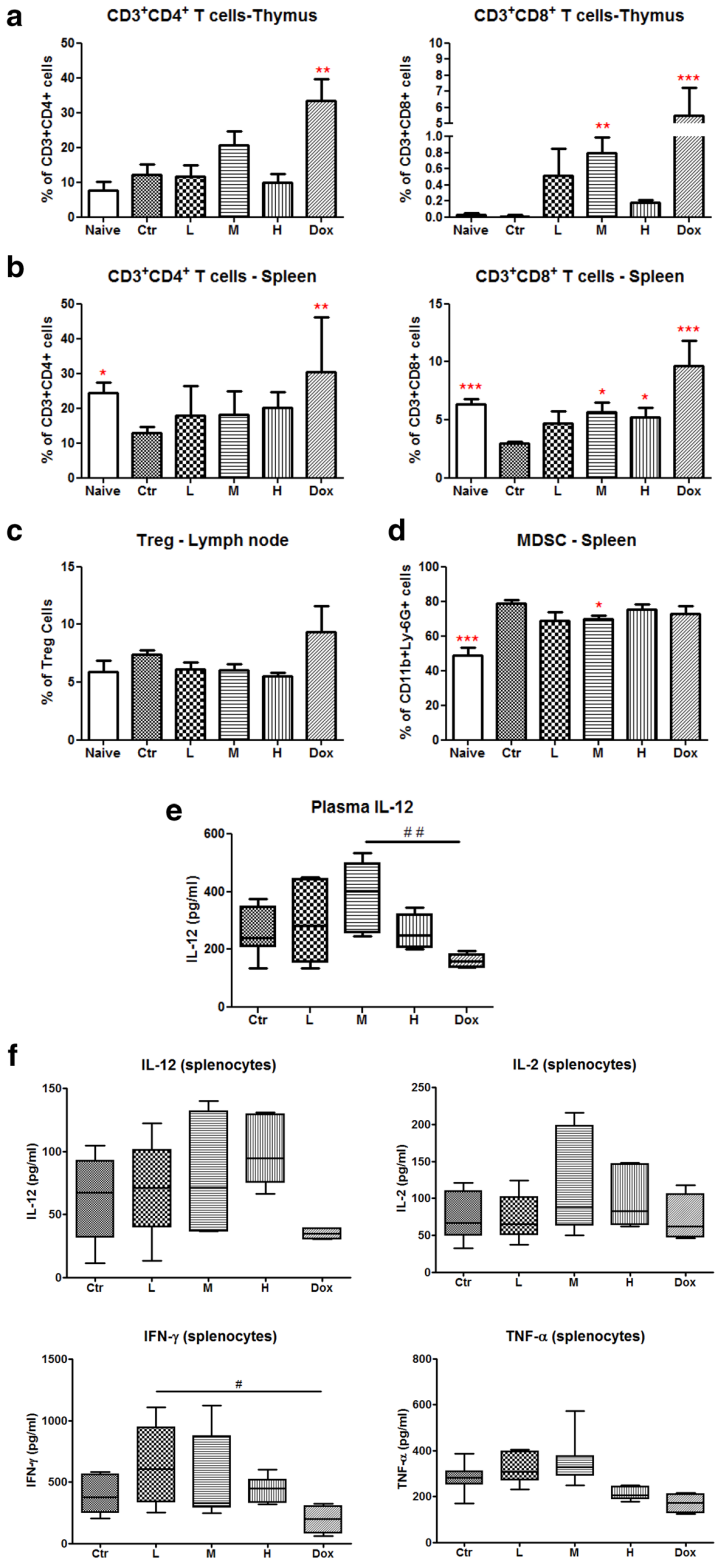
Effects of formula treatments on immune responses of tumor-bearing mice. Populations of T lymphocytes, myeloid-derived suppressor cells (MDSC) and regulatory T cells (Treg) in spleen, thymus and lymph node were determined by flow cytometry. Cells were stained with fluorochrome conjugated antibodies as mentioned in Materials and Methods section. **a**, **b** The proportions of CD4^+^ and CD8^+^ T cells in lymphocytes were analyzed using flow cytometry analyses by gating CD3^+^ population. Populations of **c** regulatory T cells (CD4^+^CD25^+^FOXP3^+^) in lymph node and **d** MDSC (CD11b^+^Ly6G^+^) in spleen were analyzed from the 10,000–20,000 viable isolated cells. Results were expressed as mean concentration ± SEM (n = 4–8). **e** Level of IL-12 in plasma of mice from different treatment groups were determined by ELISA. **f** Production of IL-2, IL-12, IFN-γ and TNF-α by isolated spleen lymphocytes from formula-treated mice. The concentrations of cytokines were determined by ELISA. Results were expressed in box and whisker plots of 4–8 mice each group. Statistical difference was determined by one-way ANOVA, followed by Tukey’s multiple comparisons test. *p < 0.05, **p < 0.01, ***p < 0.005 as compared with the control group. ^#^*p* < 0.05, ^##^*p* < 0.01 when compared among treatment groups

Since one of the component herbs within the formula possess potent immuno-stimulating activities, the overall modulatory effects of the formula were further determined. Immune cells of immunocompetent tumor-bearing mice will play crucial roles in tumor progression and hence, the effects of formula treatment on the populations of various immune cells were studied. As shown in Fig. 4a, b, CD8^+^ T cell population in thymus and spleen in formula dosage M, as well as doxorubicin groups were significantly increased when compared with the untreated control group (*p* < 0.05). Besides, the interference from regulatory T cells (Treg cells) and myeloid-derived suppressor cells (MDSC) on the activation of T cells against cancer were demonstrated in previous studies [28]. Results showed that formula treatment (especially at dosage M) could reduce the populations of Treg cells (CD4^+^CD25^+^FOXP3^+^ cells) in lymph node and MDSC (CD11b^+^Ly6G^+^ cells) in spleen (Fig. 4c, d).

On the other hand, the plasma IL-12 level in formula (at dosage M) treatment group was shown to be higher than that of control and doxorubicin-treated groups (Fig. 4e). Furthermore, spleen lymphocytes were isolated from tumor-bearing mice treated with formula activated by PHA for 24 h. The concentrations of Th-1 cytokines (IL-2, IL-12, IFN-γ and TNF-α) released by spleen lymphocytes ex vivo were higher in those from formula-treated groups than those from control group, although the differences were not statistically significant (Fig. 4f). In contrast, the levels of these cytokines were not increased in doxorubicin-treated group. These results suggested that the immune responses against tumor could be modulated by formula treatment.

### Herbal formula treatment significantly prolonged survival of breast tumor-bearing mice

**Fig. 5:**
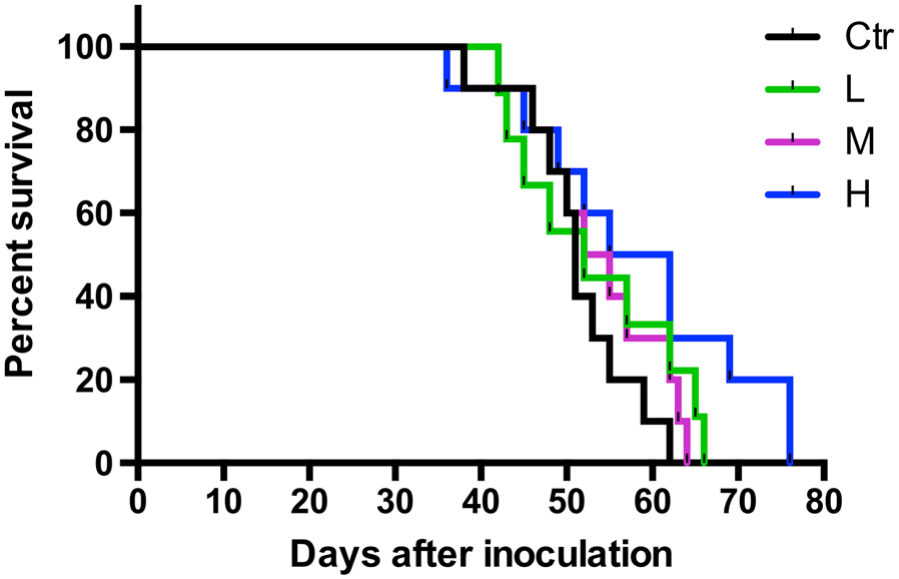
Survival curves of mice in each treatment group (n = 10). Data were shown in Kaplan–Meier survival curves

The survival rate of mice in the treatment groups were further studied. The death number of mice was recorded in either natural death from tumor-bearing or euthanasia due to the large tumor (diameter exceeded 15 mm). As shown in Fig. 5, the mice treated with formula (at dosage H) lived for the longest period as there were still 20% of mice alive until 76 days after tumor cell inoculation. Median survival days of mice in formula dosage H group was 58.5 days, which was longer than that in control (51 days) group. These results showed that formula treatment could prolong the median survival time (or increased the life span) of the metastatic breast tumor-bearing mice.

### Combined treatment of herbal formula with chemotherapeutic agent exhibited better anti-tumor effects

**Fig. 6:**
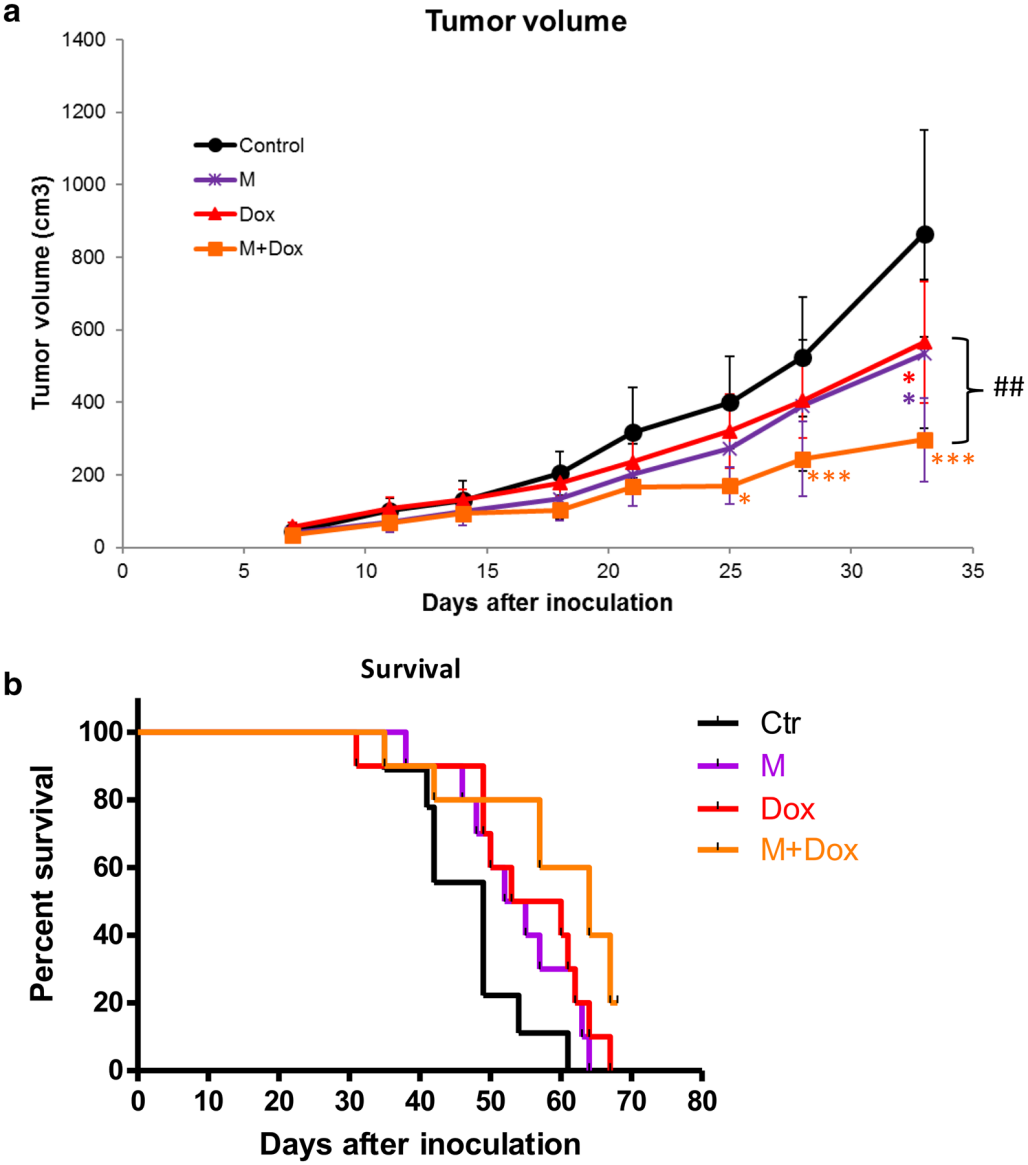
Effects of combined formula and doxorubicin (Dox) treatments on the mouse breast tumor growth and the survival of tumor-bearing mice. **a** Tumor volume in different treatment groups. Each point represented mean of 10 mice. Results were expressed as mean + SEM (n = 10). *p < 0.05, ***p < 0.005 when treatment groups compared with control group; ^##^*p* < 0.01 when compared among treatment groups. **b** Survival curves of mice in each treatment group (n = 10). Data were shown in Kaplan–Meier survival curves

Further investigation on the combined effect of herbal formula and chemotherapeutic agent was conducted in the 4T1 metastatic breast tumor-bearing mice. Mice were administered with herbal formula at the M dosage (770 mg/kg, orally daily) plus doxorubicin (5 mg/kg, intraperitoneally weekly) for 4 weeks. As shown in Fig. 6a, the tumor volume of combined treatment group was significantly lowered than that of control group from the 25th day after inoculation. The final tumor volume of combined treatment group was significantly lowered than that of doxorubicin alone treatment group (*p* < 0.01) and control group (*p* < 0.001). In the other set of experiment, the survival rate of combined treatment group was higher than those of control and doxorubicin alone treatment group (Fig. 6b). The median survival of control, formula at M dosage alone, doxorubicin alone and combined treatment groups were 49, 54.5, 56.5 and 64 days, respectively.

## Discussion

In this study, the efficacies in terms of anti-tumor, anti-metastasis, immuno-modulating and bone protective activities of the herbal formula have been evaluated in metastatic breast tumor mouse model. The composition of the herbal formula was based on our hypothesis that combining herbs with the above-mentioned bioactivities to observe synergistic effect. As shown in the results, the herbal formula exhibited remarkable bioactivities in the breast tumor-bearing mice, which was demonstrated for the first time.

As we have been suggesting to develop a novel Chinese herbal formula for multi-target therapy approaches for years [9], the efficacies of various anti-cancer herbs have been examined in our in vivo platforms as mentioned above. Here we demonstrated that treatment with two dosages (770 and 2310 mg/kg) of herbal formula resulted in tumor growth and metastasis inhibition in metastatic breast tumor-bearing mice. In fact, higher dosages (i.e. > 2310 mg/kg) have been tested in our pilot study; however, the efficacy of 7000 mg/kg (threefold of the highest dosage in present study) of formula reached plateau and the solubility of the herbal formula powder was not good for the higher dosage (i.e. ≥ 7000 mg/kg). Hence, the activities of the herbal formula at 231, 770 and 2310 mg/kg were reported here.

Metastatic breast cancer is a heterogeneous disease that has a variety of different clinical conditions [24]. It remains a major challenge in cancer treatment that the median survival is 2–3 years [25]. The development of our novel herbal formula aimed to provide an alternative/adjuvant choice for breast cancer patients. To provide pre-clinical evidences of efficacy, the metastatic breast tumor-bearing mouse model with spontaneous metastases to lungs, livers, and bones [26] was adopted. Another characteristic of metastatic breast cancer is the high incidence of bone metastases causing significant morbidity including pain, fracture and spinal cord compression [29]. Recent survey data showed that 62.5% of patients with initial metastatic breast cancer had bone involvement [30]. These patients may require additional medical care from orthopaedics specialists, other than receiving anti-cancer treatments alone. In this regards, multi-targeted anti-metastatic agents which also target for bone metastasis are desired. The bone protective activities showed in the present study could certainly provide a rationale of clinical use of the herbal formula.

On the other hand, the systemic effects of the herbal formula, including immunomodulation, could be evaluated in this syngeneic tumor-bearing model. Our results showed that the herbal formula was effective in inhibiting the metastasis to lungs and livers, which may be contributed by the potent anti-metastatic effects of AP and CS. In addition, herbal formula could modulate the CD8^+^ T cells, regulatory T cells and MDSC population as well as increase the levels of anti-tumor cytokine IL-12 (in plasma and secreted by splenocytes) [31]. The outcome of herbal formula treatment was the prolong life span of the metastatic breast tumor-bearing mice.

Furthermore, in our previous review on the role of Chinese herbal medicines in cancer management, we suggested that the potential beneficial adjuvant therapies can be attained by combining CHM and chemotherapeutics [9]. The superior anti-tumor and life prolonging effects of the combined herbal formula and chemotherapeutic treatment have demonstrated here. The synergistic effects of the combined treatment as well as the efficacies of the formula in other types of cancer are worth further investigations.

Nowadays, the use of herbal medicines by patients as adjuvant to conventional cancer treatment is common while there were concerns from the users on the efficacy and safety. Some of the herbal prescriptions or supplements for cancer patients are short of evidence-based confirmation of efficacy. In addition, their multi-targeted efficacy and bioavailability may be virtually non-existing. We aimed to apply innovative concepts of evidence-based, multi-targeted and quality-assured for developing a herbal formula targeting on metastatic breast cancer. Our findings provided scientific evidences on this hypothetically created herbal formula in breast tumor-bearing mice. We are ready to go for a clinical trial using this herbal formula and the promising outcome from the trial may provide alternative treatment option for metastatic breast cancer patients in the future.

## Conclusions

The innovative herbal formula was shown to possess anti-tumor, anti-metastatic and bone protective effects in metastatic breast tumor animal model, which would lead to the future development of adjuvant therapy for metastatic breast cancer.

## Additional file

**Additional file 1.** Minimum standards of reporting checklist.
